# Surfactant-free synthesis of fluorescent platinum nanoclusters using HEPES buffer for hypochlorous acid sensing and imaging†

**DOI:** 10.1039/d1ra09064j

**Published:** 2022-04-04

**Authors:** Xiaoying Wang, Yusong Wang, Liping Yin, Qiang Zhang, Shaozhen Wang

**Affiliations:** AnHui Provincial Engineering Research Center for Polysaccharide Drugs and Institute of Synthesis and Application of Medical Materials, Department of Pharmacy, Wannan Medical College Wuhu 241002 P. R. China wangshaozhen@wnmc.edu.cn; Laboratory of Functionalized Molecular Solids, Ministry of Education, Anhui Key Laboratory of Chemo/Biosensing, College of Chemistry and Materials Science, Anhui Normal University Wuhu 241002 P. R. China qzhang@ahnu.edu.cn

## Abstract

A surfactant-free synthesis of noble-metal nanoclusters (NMNCs) with specific function has recently remained more attractive and superior in bio-applications. Herein, by employing the weak reducibility of non-toxic HEPES, we prepared novel water-soluble fluorescent HEPES@Pt NCs by a simple surfactant-free synthesis strategy for hypochlorous acid (HClO) sensing. The as-prepared Pt NCs featured ultra-small size (∼2 nm), bright blue fluorescence, high stability and biocompatibility, and the fluorescence of the Pt NC nanoprobe can be specifically quenched with hypochlorous acid by a static quenching process. Moreover, the surfactant-free Pt NC probe displays fascinating performances for HClO sensing, including fast response to HClO, high stability and specificity, and is further applied for imaging the fluctuations of the HClO concentration in living cells with satisfactory results for the first time. Thereby, we anticipate that it is a reliable and attractive approach to develop versatile NMNCs through the surfactant-free synthesis for further applications in biological research.

## Abstract 2

In this study, by employing the weak reducibility of non-toxic HEPES, a novel water-soluble fluorescent HEPES@Pt NC was successfully prepared by a simple surfactant-free synthesis strategy for ClO^−^ sensing and imaging.

## Graphical abstract

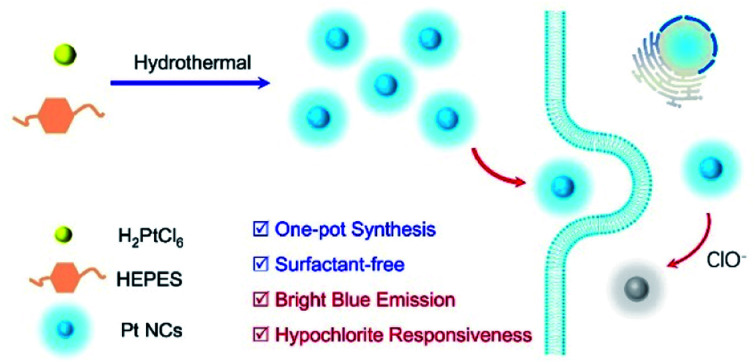

## 1. Introduction

Noble-metal nanoclusters (NMNCs) composed of several to tens of atoms, as attractive engineering nanomaterials, have promisingly emerged with versatile applications in energy,^1^ catalysis,^2^ medicine,^3^ environment,^4^ fluorescent labeling,^5^ biosensing^6^ and bioimaging^7^ due to their unique physicochemical properties, including chemical stability, size-dependent properties, photoluminescence and surface effects.^8–10^ Among them, platinum nanoclusters (Pt NCs) as typical NMNCs^11^ along with their high stability, excellent luminescent performances and ultra-small sizes in the fields of fluorescent labeling, biosensing and bioimaging make them more attractive and superior.^12–14^ For the first time, Inouye *et al.* rationally designed and fabricated a water-soluble, blue-emitting Pt NC-based fluorescent probe for applications in fluorescence imaging in living cells.^15^ Since then, Pt NCs were gradually exploited as the basic nano-frameworks to design and prepare fluorescent probes for biosensing and bioimaging.^16–21^ Surfactants and high molecular polymers are generally introduced into the synthesis process of Pt NCs as stabilizers to control their sizes and prevent the aggregation of nanoparticles.^22,23^ However, these surfactants and polymers are inevitably adsorbed onto the surface of the nanoclusters and may decrease activity and increase toxicity, which is negative for bioimaging and other biological applications.^24,25^ In order to overcome the negative effects derived from the surfactants, the surfactant-free synthetic strategy has been developed, and the derived surfactant-free NMNCs possess enhanced performances, including catalytic activity and biocompatibility.^26–34^ Thereby, it is an attractive way to prepare the Pt NCs with desired bio-function through a surfactant-free synthesis.

In addition, hypochlorite/hypochlorous acid (ClO^−^/HClO), as a crucial ROS (reactive oxygen species), has been widely used in daily life for antibacterial and disinfecting viruses.^35,36^ More importantly, HClO/ClO^−^ plays a vital role in mediating numerous physiological and pathological activities in the biosystem.^37,38^ Endogenous hypochlorite can be generated through the myeloperoxidase (MPO)-catalyzed peroxidation of chloride ions (Cl^−^) using hydrogen peroxide (H_2_O_2_), and it served as a crucial innate immune defense killing invading bacteria and pathogens.^39,40^ Moreover, excessive hypochlorite can induce cellular oxidative stress and lead to the denaturation of proteins, enzymes, lipid droplets and nucleic acids through oxidation or chlorination, which directly causes tissue damage and is tightly related to neurodegenerative disorders,^41^ inflammatory diseases,^42,43^ liver injuries^44^ and even cancer.^45^ Taking into account the above scientific issues, it is essential and urgent to develop a surfactant-free NMNC-based fluorescent probe with high biocompatibility to monitor hypochlorous acid in biosystems.

**Scheme 1:**
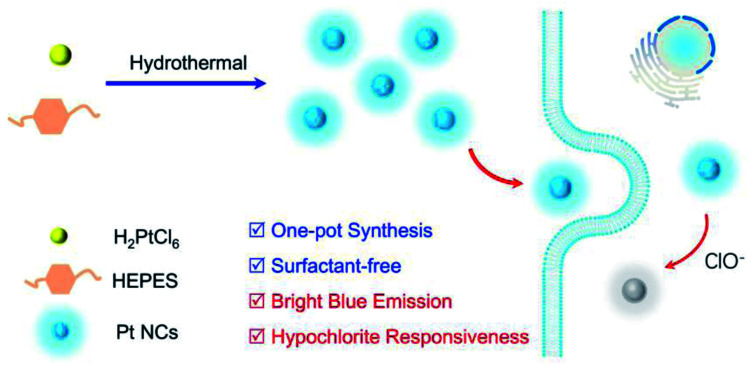
Schematic of a one-pot surfactant-free synthesis of fluorescent Pt NC for hypochlorous acid imaging in living cells.

Herein, by employing the appropriate reducibility of 2-[4-(2-hydroxyethyl)piperazin-1-yl]ethanesulfonic acid (HEPES),^46^ a surfactant-free, ClO^−^-responsive blue-emitting Pt NCs were successfully fabricated by a one-pot hydrothermal method.(Scheme 1)

The non-biotoxicity of HEPES endowed the obtained Pt NCs with high biocompatibilities, which were very beneficial for the biological application of the Pt NCs. Moreover, the as-prepared surfactant-free Pt NCs displayed satisfactory performances, including fast response rate, high stability and favorable selectivity towards ClO^−^ sensing, and were further successfully applied to image endogenous and endogenous ClO^−^ in living cells. Taken together, this work not only developed a surfactant-free Pt NC-based fluorescent probe for sensing and imaging ClO^−^, but also provided some remarkable perspectives for the surfactant-free synthesis of noble-metal nanoclusters.

## 2. Experimental

### 2.1 Procedures for the synthesis of fluorescent Pt NCs

Fluorescent Pt NCs were fabricated by a one-pot hydrothermal approach. In particular, H_2_PtCl_6_·6H_2_O (10 mM, 1 mL), 12 mL deionized water and HEPES buffer (1 M, 2 mL) were added into a Teflon-lined stainless-steel autoclave. Subsequently, the reactor was heated at 160 °C for 4 hours. The resulting solution was first filtered through a 0.1 μM membrane filter and centrifuged to remove the agglomerates and large nanoparticles. Then, the solution was purified by dialysis using a dialysis bag (500–1000 Da, molecular weight cutoff) for 48 h, and the dialysis water was replaced with fresh deionized water every 6 h during this period. The obtained Pt NC colloidal solution was stored at 4 °C for further use.

### 2.2 Procedures for ClO^−^ sensing

Typically, 500 μL of a mixture of Pt NCs (10 μg mL^−1^) and ClO^−^ at different amounts was reacted in PBS buffer (10 mM, pH 7.4) for 10 min at 37 °C. After the completion of the reaction, the fluorescence spectrum was recorded at an excitation wavelength of 360 nm.

### 2.3 Imaging of ClO^−^ in living cells

For fluorescent imaging experiments in living cells, the HeLa cells in the exponential growth phase were first seeded in confocal dishes and incubated to adhere for 24 h under a humidified atmosphere containing 5% CO_2_. After the adherence of the cells to the dishes, the HeLa cells were incubated with fresh 1640 medium containing 10 μg mL^−1^ Pt NCs for 8 h. For imaging exogenous ClO^−^, the Pt NC-stained cells were treated with 100 μM ClO^−^ for 30 min. For imaging endogenous ClO^−^, the cells were pre-incubated with PMA (4 μg mL^−1^) and LPS (1 μg mL^−1^) for 0.5 h and 4 h, respectively. Then, the cells were further cultured with Pt NCs for 8 h. In addition, for the control of endogenous experiments, the cells were treated with PMA (phorbol 12-myristate 13-acetate, 4 μg mL^−1^), LPS (lipopolysaccharide, 1 μg mL^−1^) and NAC (*N*-acetyl-l-cysteine, 1 mM) for 0.5 h, 4 h and 2 h, respectively. Then, the treated cells were further cultured with Pt NCs for 8 h. All of the fluorescence images were recorded in the range of 420–550 nm through a confocal laser scanning microscope at an excitation of 405 nm.

## 3. Results and discussion

### 3.1 Preparation and characterization of the Pt NCs

**Fig. 1:**
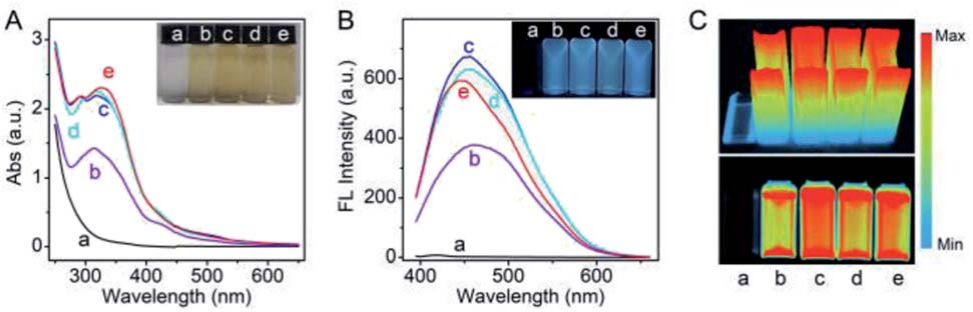
The absorption spectrum (A) and the fluorescence spectrum (B) at different reaction times of 0.5 h (a), 2.0 h (b), 4.0 h (c), 5.5 h (d) and 7.0 h (e). (C) Fluorescence images (bottom panel) and the 3D intensity images (top panel) at different reaction times.

In this study, in order to achieve the desired surfactant-free Pt NCs with enhanced biocompatibility, HEPES was employed as the suitable reducing agent to fabricate the fluorescent Pt NCs. HEPES is commonly used as a well buffer in biochemical experiments due to its non-toxic and good buffering capacity at working concentration, and its weak reducing agent properties were confirmed and employed to synthesize precious metal nanoparticles. Therefore, the surfactant-free Pt NCs were synthesized using H_2_PtCl_6_·6H_2_O and HEPES as the precursor and reducing agent, respectively, by a one-pot hydrothermal method. During the reaction, the solution changed its color from light yellow (0 h) (Fig. S1A†) to colorless (0.5 h), and finally to yellow (4–7 h) (the inset of Fig. 1A). As shown in Fig. 1A, the UV absorption between 260 and 600 nm gradually increases during the reaction and finally remains stable, indicating that PtCl_6_^−^ gradually reduced and formed Pt NCs. As described in Fig. 1B and C, the fluorescence brightness of the Pt NCs prepared with different times is also investigated, and the results suggest that the brightest Pt NCs can be achieved by reacting for 4 h. Therefore, the Pt NCs reacted for 4 h were assessed as the best candidate nanoprobes for subsequent sensing and imaging experiments.

**Fig. 2:**
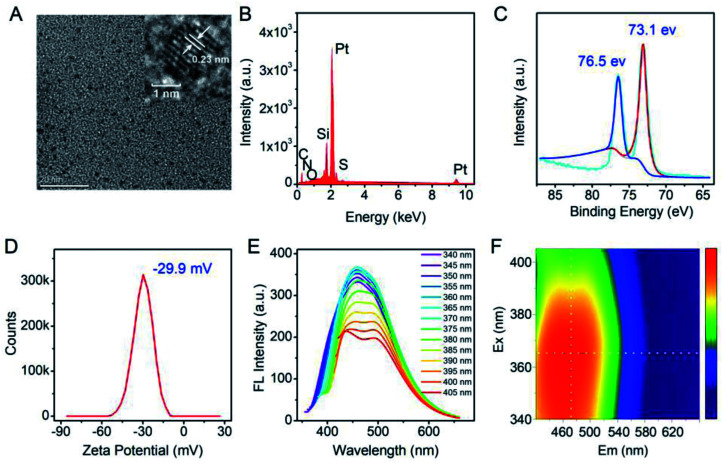
(A) TEM and HRTEM (inset) images of the surfactant-free Pt NCs. (B) EDS elemental analyses of the Pt NCs on the silicon substrate. (C) X-ray photoelectron spectra (XPS) of the Pt 4f region of the Pt NCs. (D) zeta potential of the Pt NCs. (E) The fluorescence emission spectra of the Pt NCs at an excitation in the range of 340–405 nm. (F) Excitation–emission matrix of the Pt NCs.

After purification, TEM, EDS and XPS were employed to characterize the Pt NCs. From Fig. 2A, it can be observed that the TEM image of the proposed Pt NCs exhibits numerous small and uniform nanostructure with a diameter of ∼2 nm, and the HRTEM image indicates that a lattice spacing of the proposed Pt NCs is 0.23 nm, which is consistent with the (111) facets of Pt. Furthermore, these nanoparticles were confirmed to be the platinum species by the EDS analysis (Fig. 2B). The XPS Pt 4f displayed two binding energies at 73.1 and 76.5 eV, which are attributed to the Pt 4f_7/2_ and Pt 4f_5/2_ peaks, respectively, suggesting that the obtained nanoclusters were the zero valent Pt. Furthermore, the optical properties of the Pt NCs were studied, and the results suggested that the optimal excitation wavelength of the resultant Pt NCs with blue fluorescence emission centered at 470 nm was 365 nm in PBS buffer (10 mM, pH 7.4). Considering the above results of the characterization together, the surfactant-free fluorescent Pt NCs were successfully synthesized through the proposed HEPES reduction method. In addition, the zeta potential of the Pt NCs was measured to be −29.9 eV, indicating that the surface of the Pt NC was negatively charged and possessed a good colloidal stability.

### 3.2 HClO sensing and its quenching mechanism

**Fig. 3:**
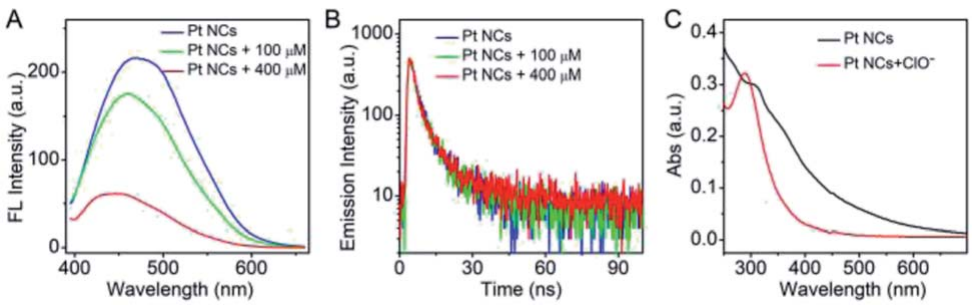
(A) Fluorescence emission spectra of Pt NCs (blue curve), Pt NCs with 100 μM ClO^−^ (green curve) and Pt NCs with 400 μM ClO^−^ (red curve). (B) Time-resolved fluorescence decays of Pt NCs (blue curve), Pt NCs with 100 μM ClO^−^ (green curve) and Pt NCs with 400 μM ClO^−^ (red curve) in pure water. (C) The UV-vis absorption spectra of the Pt NCs (black curve) and the Pt NCs with 400 μM ClO^−^ (red curve).

The feasibility of the Pt NCs in the detection of ClO^−^ was investigated. As represented in Fig. 3A, the Pt NCs exhibit a strong fluorescence emission at 470 nm under the excitation of 365 nm in PBS buffer (blue curve). When ClO^−^ was introduced into the Pt NC solution, the fluorescent emission of Pt NCs was dramatically quenched (Fig. 3A, green curve), and more fluorescence quenching was observed with increased ClO^−^ concentrations (Fig. 3A, red curve). The feasibility experiment indicated the oxidation quenching process of the Pt NCs with the addition of ClO^−^, and it could be further applied for ClO^−^ sensing. To investigate the oxidation quenching process between the Pt NCs and ClO^−^, the fluorescence lifetimes of the Pt NCs and Pt NCs treated with ClO^−^ were measured. As shown in Fig. 3B, the fluorescence lifetime of the Pt NCs is found to be 6.08 ns, while the fluorescence lifetime of the Pt NCs with the addition of ClO^−^ remained almost unchanged (6.06 ns), suggesting the static quenching process mediated by the ClO^−^ oxidation of Pt NCs. In addition, the UV-vis absorption spectra display a significant change after the addition of ClO^−^, which further confirmed the static quenching process. Taking these results together, the quenching of the fluorescent Pt NCs by ClO^−^ is based on the oxidation quenching mechanism with a static quenching process.

### 3.3 Analysis performances of the Pt NCs for HClO sensing

**Fig. 4:**
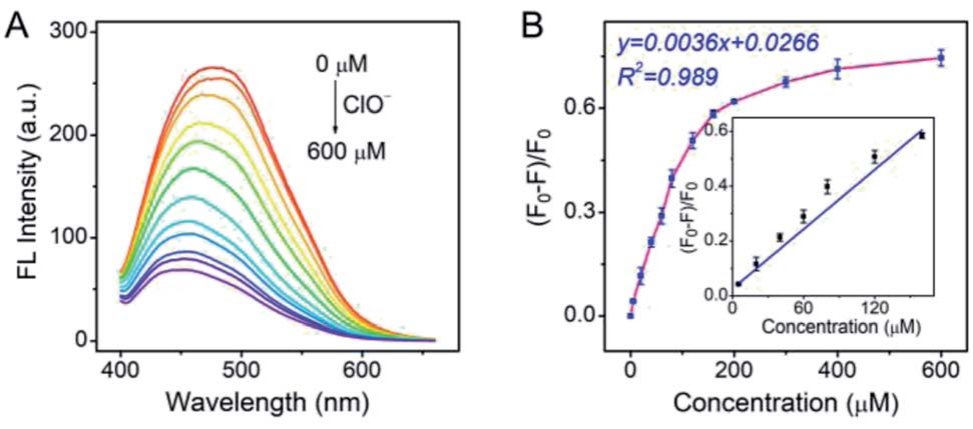
(A) Fluorescence emission spectra of the Pt NCs in PBS buffer with different ClO^−^ concentrations. (B) Calibration curve of (*F*_0_ − *F*)/*F*_0_ against different ClO^−^ concentrations for the Pt NCs. *F*_0_ and *F* are the intensities at 470 nm of Pt NCs in the absence and presence of ClO^−^, respectively.

As the Pt NCs displayed the potential capabilities of ClO^−^ sensing, the sensing performances of the Pt NCs were investigated in detail. As shown in Fig. 4A, the fluorescence intensities of the Pt NCs at 470 nm dramatically attenuated with increased ClO^−^ concentrations, which is attributed to the static quenching process between the Pt NCs and ClO^−^. A good linear relationship between the (*F*_0_ − *F*)/*F*_0_ and the ClO^−^ concentrations in the range of 5–160 μM was obtained with a correlation coefficient of 0.989 and a sensitivity of 0.0036 μM^−1^ (Fig. 4B). The limit of detection (LOD) was also calculated to be 1.8 μM, which was sufficient to probe the ClO^−^ concentration in living cells.

**Fig. 5:**
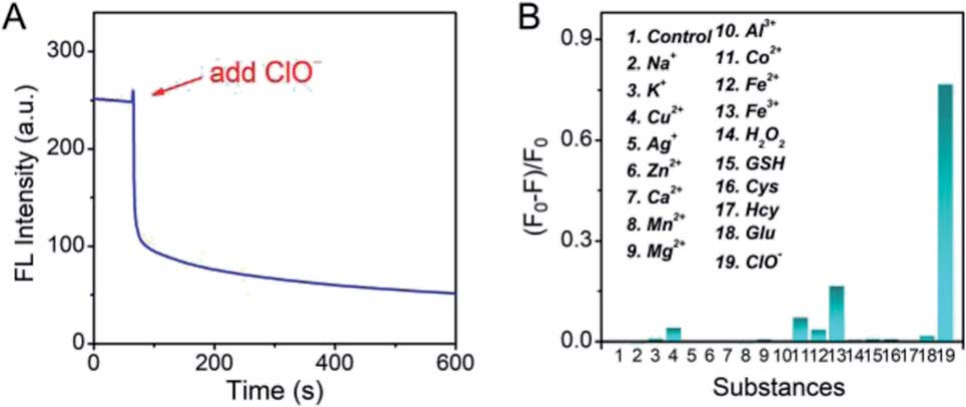
(A) Time-dependent fluorescence intensities at 470 nm for the Pt NCs upon addition of ClO^−^. (B) The selectivity of the Pt NC fluorescent probe to different potential interfering substances in PBS buffer (ClO^−^: 500 μM; H_2_O_2_: 2 mM; GSH: 2 mM; the concentration of other interfering substances is 100 μM).

To further study the performances of Pt NCs as fluorescent nanoprobes for ClO^−^ sensing, the fluorescence response time curve was first measured. From Fig. 5A, it can be seen that the Pt NCs display a fast response within 5 min towards ClO^−^ in PBS buffer (10 mM, pH 7.4). Subsequently, the selectivity of the Pt NC-based nanoprobe for detecting ClO^−^ against potential interfering substances, including metal ions, ROS (reactive oxygen species) and thiol-containing substances, was studied. As shown in Fig. 5B, the fluorescent intensity of the Pt NC at 470 nm is difficult to be quenched by other potential interfering substances, indicating that the proposed Pt NCs featured high selectivity and can serve as a nanoprobe for ClO^−^ analysis in biosystems. Moreover, in order to study the stability of the Pt NC-based probe in detail, the experiments, including pH-resisting stability (Fig. S2†), the effect of ionic strength (Fig. S3†) and photostability (Fig. S4†) of the Pt NC, were also carried out. As described in Fig. S2,† the Pt NCs display stable fluorescence in a wide pH range, indicating that the Pt NCs featured high resistances to the effect of pH. Fig. S3† demonstrates that the fluorescence emissions of Pt NCs are hardly affected by the ionic strength (*I*). The photostability of the Pt NCs was investigated by measuring the attenuation of the fluorescence intensities of the Pt NCs at 470 nm under a continuous excitation at 405 nm for 60 min. It can be seen from Fig. S4† that the intensities at 470 nm remain unchanged after 60 min of excitation, indicating that the Pt NCs possess excellent photostability. All of the above results demonstrated that the proposed Pt NCs possessed excellent ClO^−^ analysis performance and stability. When compared with the other nanoprobes of ClO^−^ (Table S1†), the proposed Pt NC-based fluorescent probe fabricated by surfactant-free one-step synthesis displayed excellent detection performance, high stability and biocompatibility, suggesting that the Pt NCs could serve as nanoprobes for sensing and imaging in biosystems.

**Fig. 6:**
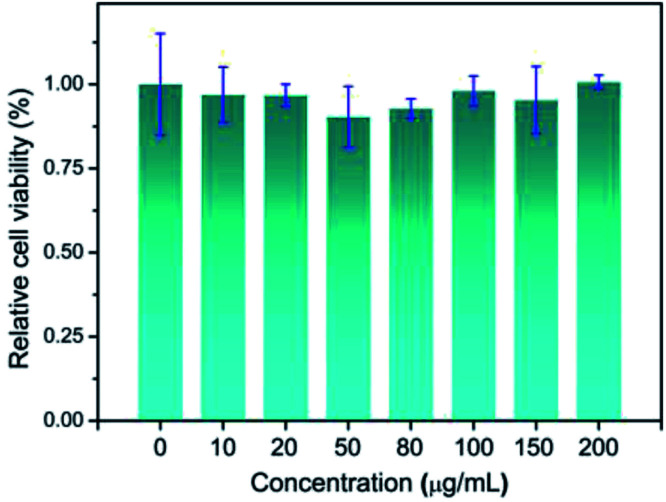
The viability of the HeLa cells treated by the Pt NCs at various concentrations for 24 h.

### 3.4 Fluorescence imaging HClO in living cells

**Fig. 7:**
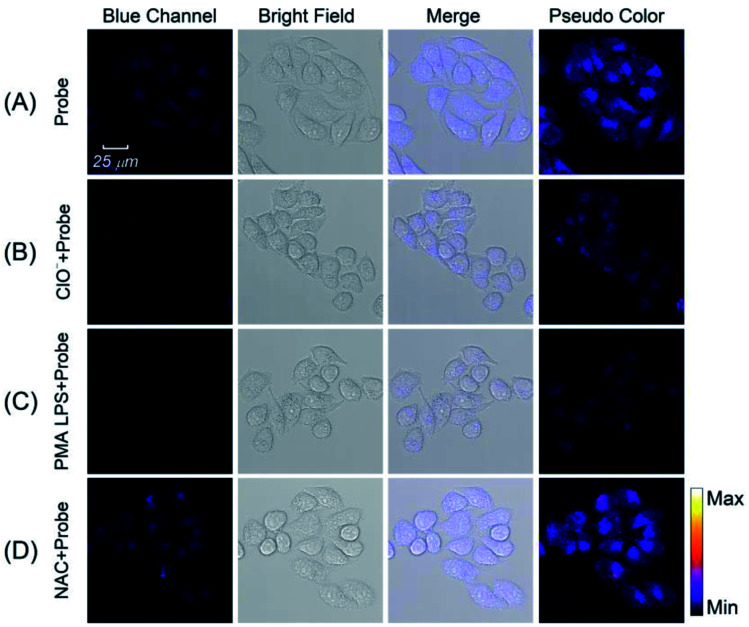
The fluorescence imaging of exogenous and endogenous ClO^−^ in living cells. (A) HeLa cells were only treated with the Pt NCs. (B) The Pt NC-stained HeLa cells were treated with 100 μM ClO^−^ for 30 min. (C) HeLa cells were pre-incubated with PMA (4 μg mL^−1^) and LPS (1 μg mL^−1^) for 0.5 h and 4 h, respectively. Then, the treated cells were further cultured with Pt NCs for 8 h. (D) HeLa cells were pre-incubated with PMA (4 μg mL^−1^), LPS (1 μg mL^−1^) and NAC (1 mM) for 0.5 h, 4 h and 2 h, respectively. Then, the treated cells were further cultured with Pt NCs for 8 h.

Motivated by the satisfied sensing performances of the Pt NCs, the capability of imaging intracellular ClO^−^ in living cells using Pt NCs as the probe was evaluated. Previously, the cytotoxicity of the Pt NCs was first studied through the standard MTT assay, and the results demonstrated that the as-prepared Pt NCs possessed very low cytotoxicity to living cells, suggesting that Pt NCs can serve as biocompatible nanoprobes for bioimaging (Fig. 6). Subsequently, the capability of the Pt NCs for the exogenous and endogenous ClO^−^ imaging of living cells was studied through confocal laser scanning microscopy (CLSM). As shown in Fig. 7A, the Pt NC-stained HeLa cells display a significant fluorescence in the blue channel, indicating that the Pt NCs possessed good cell permeability. After the Pt NC-stained cells were treated with 100 μM ClO^−^, remarkable fluorescence attenuation in the blue channel is observed (Fig. 7B), implying that the intracellular Pt NC probe could be efficiently quenched by exogenous ClO^−^. Moreover, PMA and LPS are used to stimulate the HeLa cells to generate endogenous hypochlorous acid. As shown in Fig. 7C, the living cells pretreated with PMA and LPS display a negligible fluorescence in the blue channel and pseudocolor compared with Fig. 7A, indicating that the Pt NCs can track the endogenously elevated ClO^−^ in cells. To confirm that the fluorescence quenching response was derived from the endogenous ClO^−^, a control experiment was performed by treating NAC (a free-radical scavenger) to remove endogenous ClO^−^. As expected, a strong fluorescence that resulted from the treated cells was observed, revealing that NAC scavenged the endogenously generated ClO^−^ and inhibited the oxidation quenching of the Pt NCs, as described in Fig. 7D. Compared with longer wavelength hypochlorous acid fluorescent probes, the short excitation and emission wavelengths of the proposed Pt NC nanoprobes limit their applications in *in vivo* imaging;^47^ however, on the basis of the above satisfactory experimental results, we can see that the proposed Pt NCs could serve as nanoprobes for visualizing the ClO^−^ levels in living cells.

## 4. Conclusions

In conclusion, by employing suitable reducibility and fascinating biocompatibility of HEPES, a novel surfactant-free blue-emitting Pt NC-based fluorescent probe for the detection of ClO^−^ with high stability and biocompatibility was successfully synthesized by a one-step hydrothermal reduction and further applied for imaging the fluctuations of ClO^−^ in living cells with satisfactory results. Moreover, we anticipated that it is a reliable and attractive approach to develop Pt NCs with desired bio-function through a surfactant-free synthesis for further applications in biological research.

## Conflicts of interest

The authors declare no competing financial interest.

## Supplementary Material

RA-012-D1RA09064J-s001
