# Supplementary material for: Surfactant-free synthesis of fluorescent platinum nanoclusters using HEPES buffer for hypochlorous acid sensing and imaging
Source: RSC Adv. 2022 Apr 4;12(17):10395–400. doi: 10.1039/d1ra09064j (PMC8978884; doi:10.1039/d1ra09064j)
Supplement: RA-012-D1RA09064J-s001 [file RA-012-D1RA09064J-s001.pdf]

Electronic Supplementary Material (ESI) for RSC Advances.
This journal is © The Royal Society of Chemistry 2022

Supplementary Information

Surfactant-Free Synthesis of Fluorescent Platinum Nanocluster Using HEPES Buffer for Hypochlorous Acid Sensing and Imaging

Xiaoying Wang[b], Yusong Wang[a], Liping Yin[a], Qiang Zhang[b] and Shaozhen Wang*[a]*

[*] X. Wang, Y. Wang, L. Yin, Q. Zhang, and Prof. Dr. S. Wang

[a]Anhui Provincial Engineering Research Center for Polysaccharide Drugs and Institute of Synthesis and Application of Medical Materials, Department of Pharmacy, Wannan Medical College, Wuhu 241002, P.R. China.

[b]Laboratory of Functionalized Molecular Solids, Ministry of Education, Anhui Key Laboratory of Chemo/Biosensing, College of Chemistry and Materials Science, Anhui Normal University, Wuhu 241002, P. R. China.

*Corresponding author. *E-mail*: wangshaozhen@wnmc.edu.cn.

Reagents and Instrumental

NaCl (AR, ⩾99.5%), KCl (AR, ⩾99.5%), $AgNO_3$ (AR, ⩾99.8%), $CuCl_2·2H_2O$ (AR, ⩾99.0%), $ZnSO_4·7H_2O$ (AR, ⩾99.5%), $CaCl_2·2H_2O$ (AR), $MnSO_4·H_2O$ (AR, ⩾99.0%), $MgSO_4$ (AR, ⩾98.0%), $AlCl_3·6H_2O$ (AR, ⩾97.0%), $CoSO_4·7H_2O$ (AR, ⩾99.5%), $FeSO_4·7H_2O$ (AR), $FeCl_3·6H_2O$ (AR) and H_2O_2 (AR, ⩾30%) were purchased from Sinopharm Chemical Reagent Co., Ltd. (China). Glutathione (GSH), cysteine (Cys), homocysteine (Hcy), glucose, sodium hypochlorite solution (reagent grade, available chlorine 4.00-4.99%), chloroplatinic acid hexahydrate (ACS reagent), Phorbol 12-myristate 13-acetate (PMA), *N*-acetyl-L-cysteine (NAC) and lipopolysaccharide (LPS) were purchased from Sigma-Aldrich Co. Ltd. (U.S.A.). HEPES buffer (1 M, pH 7.2- 7.4) was purchased from Beijing Soleibao Technology Co., Ltd. All aqueous solutions were prepared by deionized water (18.25 MΩ·cm).

TEM (Transmission electron microscopic) images were obtained with a Hitachi HT 7700 TEM instrument. The fluorescence and UV absorption spectra were acquired by a Hitachi F-2500 fluorescence spectrophotometer and Hitachi U-3900 spectrophotometer, respectively. The zeta potential was obtained with a Malvern Zetasizer Nano. The representative fluorescence images were in bottles were acquired by a Fusion FX7 imaging instrument (VILBER). The confocal fluorescence images were carried out on a confocal laser scanning microscope (TCS SP8, Leica, Germany).

Table S1. Comparison of other nanoprobes for sensing of ClO^{-}.

Nanoprobes	Surface group	Linear Range (μM)	Synthesis steps	Application	References
CdSe-ZnS quantum dots	Poly-NHMe	0-0.83	Three steps	Cells imaging	*Anal. Chem.* 2010, **82**, 9775–9781.
N-doped carbon dots	GSH	60-150	One step	Cells imaging	*Analyst*, 2018, **143**, 5834-5840.
Cu NCs	PVP	0-6	Two steps	Tap water sample	*ACS Appl. Nano Mater.* 2020, **3**, 312-318.
Au NCs	BSA	0.8-800	One step	Tap water sample	*Talanta,* 2015, **132**, 790-795.
CD/CCM@ZIF-8	--	0.1-50	Two steps	Serum sample	*Anal. Chem.* **2020**, *92*, 3447-3454.
$PFOBT_{36}SeTBT_{5}$ Polymer dots	PEG	0-250	Multi-step organic synthesis	Cell imaging	*J. Am. Chem. Soc.* **2017**, *139*, 6911-6918.
MoS_2 quantum dots	GSH	5-500	One step	Tap water sample	*Microchimica Acta,* 2018, **185**, 233.
Pt NCs	Hepes (Surfactant-free)	5-160	One step	Cell imaging	This work.

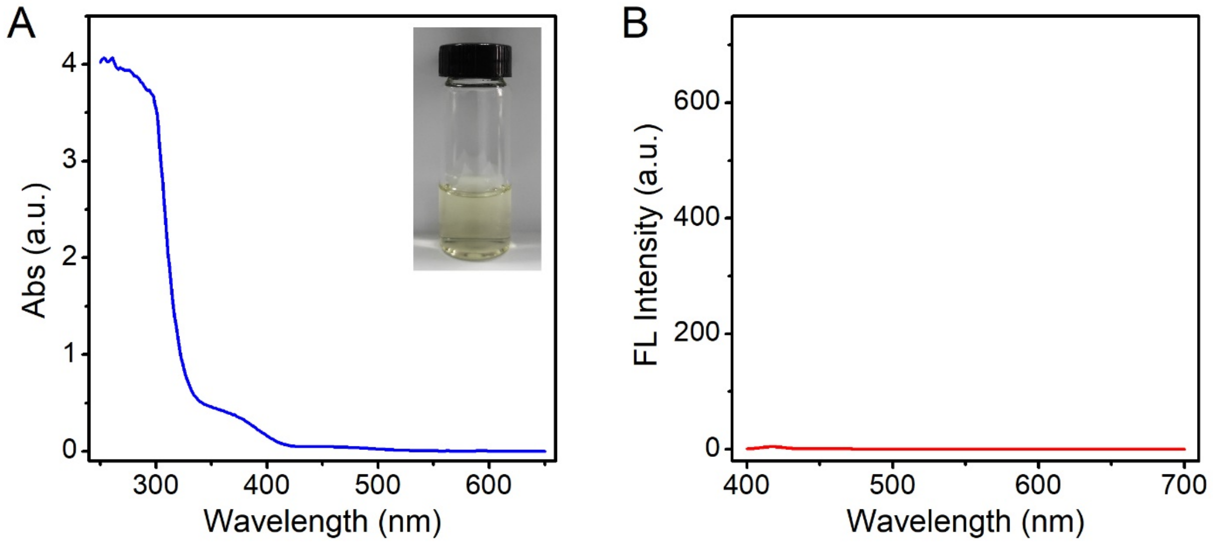

Figure S1. The absorption spectrum (A) and fluorescence spectrum (B) at 0 h reaction time.

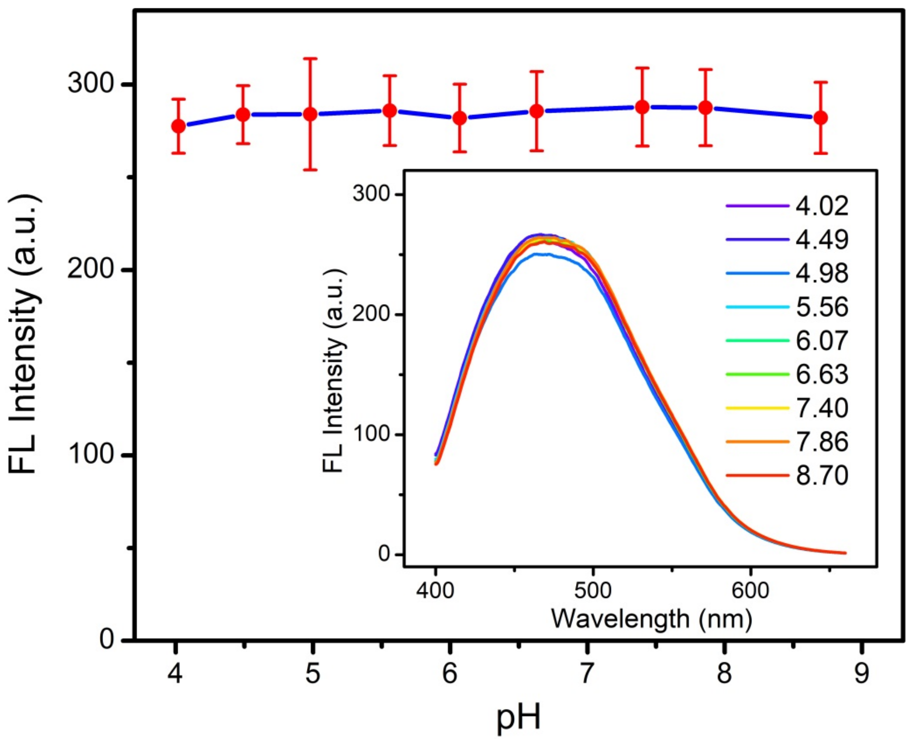

Figure S2. The relationship between the fluorescence intensity and pH value in 10 mM PBS buffer. The inset is the pH-dependent fluorescence spectra of Pt NCs.

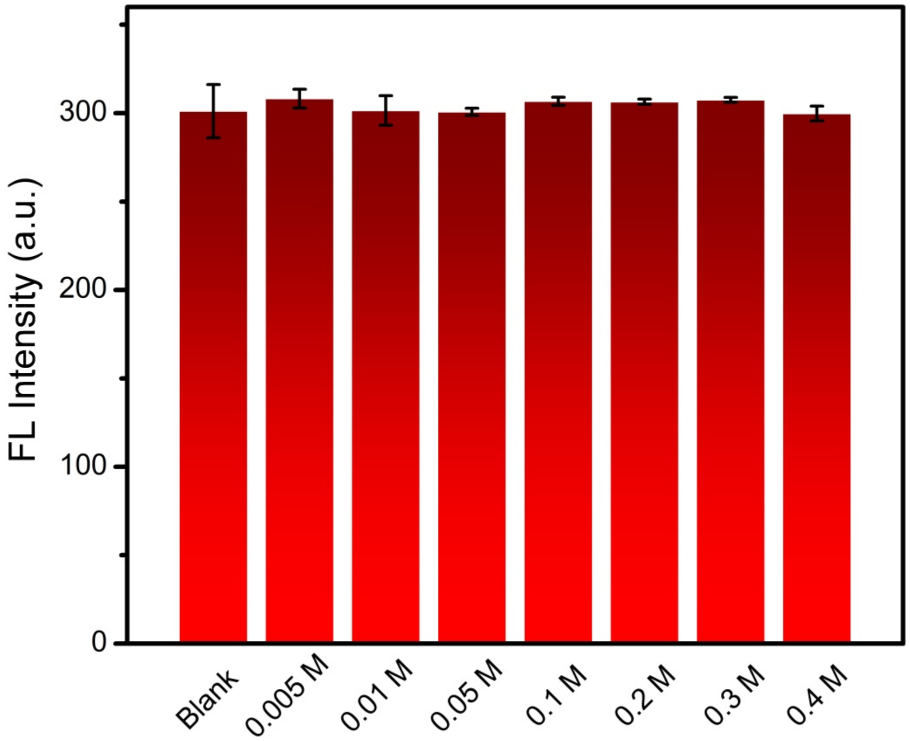

Figure S3. The dependence of ionic strength (*I*) on the fluorescence intensity of Pt NCs.

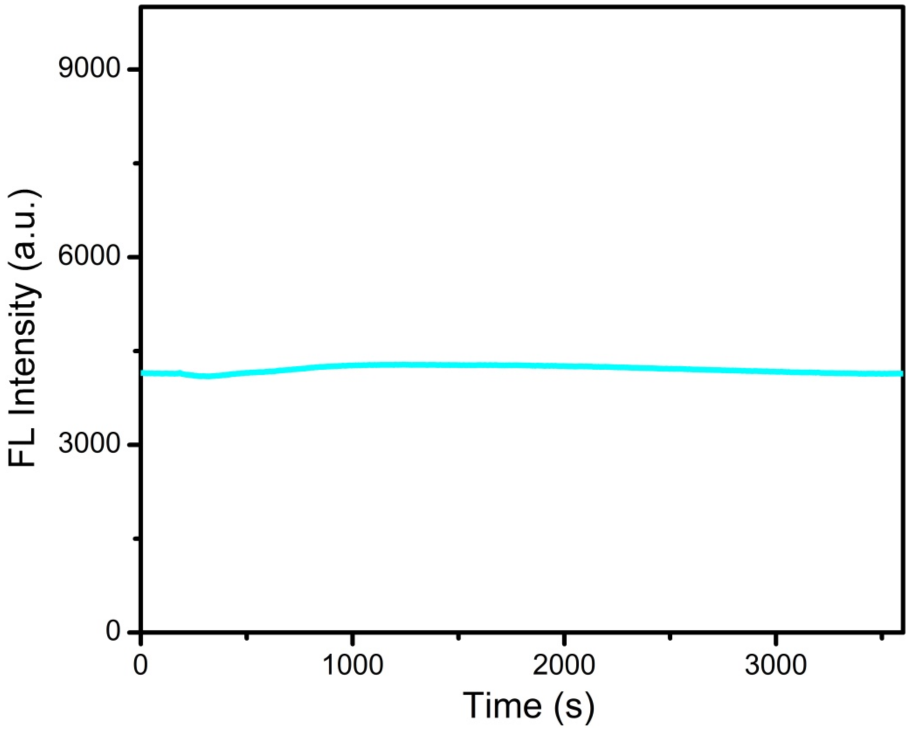

Figure S4. Photostability experiment of the Pt NCs in PBS buffer (10 mM, pH= 7.4). Voltage of xenon lamp: 700 V, slit: 15 × 15. Ex=405 nm. The concentration of Pt NCs is 10 μg/ mL.
